# Rats overexpressing the dopamine transporter display behavioral and neurobiological abnormalities with relevance to repetitive disorders

**DOI:** 10.1038/srep39145

**Published:** 2016-12-15

**Authors:** Ravit Hadar, Henriette Edemann-Callesen, Claudia Reinel, Franziska Wieske, Mareike Voget, Elena Popova, Reinhard Sohr, Yosef Avchalumov, Josef Priller, Christoph van Riesen, Imke Puls, Michael Bader, Christine Winter

**Affiliations:** 1 Department of Psychiatry and Psychotherapy, University Hospital Carl Gustav Carus, Technische Universität, Dresden, Germany; 2 International Graduate Program Medical Neurosciences, Charité Universitätsmedizin Berlin, Germany; 3 Max-Delbrück-Center for Molecular Medicine, Berlin, Germany; 4 Department of Neuropsychiatry and Laboratory of Molecular Psychiatry, Charité Universitätsmedizin Berlin, Cluster of Excellence NeuroCure, BIH and DZNE, Berlin, Germany; 5 Department of Neurology, Charité Universitätsmedizin Berlin, Germany; 6 Klinik für Psychiatrie und Psychotherapie, Charité Universit, Berlin, Germany; 7 Department of Endocrinology, Charité Universitätsmedizin Berlin, Germany; 8 Institute for Biology, University of Lübeck, Lübeck, Germany; 9 Department of Physiology and Biophysics, Federal University of Minas Gerais, Belo Horizonte, Brazil

## Abstract

The dopamine transporter (DAT) plays a pivotal role in maintaining optimal dopamine signaling. DAT-overactivity has been linked to various neuropsychiatric disorders yet so far the direct pathological consequences of it has not been fully assessed. We here generated a transgenic rat model that via pronuclear microinjection overexpresses the DAT gene. Our results demonstrate that DAT-overexpression induces multiple neurobiological effects that exceeded the expected alterations in the corticostriatal dopamine system. Furthermore, transgenic rats specifically exhibited behavioral and pharmaco-therapeutic profiles phenotypic of repetitive disorders. Together our findings suggest that the DAT rat model will constitute a valuable tool for further investigations into the pathological influence of DAT overexpression on neural systems relevant to neuropsychiatric disorders.

In the realm of neuroscience, preclinical studies promote our understanding of normal and pathological brain function as well as the development of new treatment strategies and are thus invaluable. This leads to ongoing innovation and generation of new model rodents. The development of new models is either done by the selection of existing phenomena or the rationale driven manipulation of a specific mechanism. The latter may comprise environmental, pharmacological or genetic manipulations. Genetic models start with addressing the etiology of the modeled disorder^1^ however they may only be considered complete upon meeting further construct, face and predictive validity criteria. In neuro-psychiatry etiology is mostly obscure, forcing scientists into testing the assumed etiology by comprehensively evaluating the consequences of the manipulation on aspects of brain and behavior known to be aberrant in the modeled disorder. Preclinical studies succumb to a classical differentiation between mice and rats, such that it is mostly mice, which provide rationale-driven genetic models whereas rats are devoted to behavioral and environmental manipulations, due to their superior social and behavioral repertoire. Clearly, the latter is the essence of psychiatric disorders, hence genetic rat models would ideally incorporate both aspects.

Interdisciplinary evidence suggests a pivotal role of the dopamine system and the corticostriatal circuitry^2^ in the pathology underlying repetitive disorders. Reduced tonic extracellular^3^, increased presynaptic^4^, and pharmacologically released intrasynaptic dopamine contents^5^ as well as increased dopamine receptor availability^6^, suggests an overactive dopamine transporter (DAT)^7^,^8^ in repetitive disorders, including Tourette syndrome (TS). Still, investigations into the direct consequences of DAT overexpression is underrepresented in preclinical studies with only very few models that allow insights into its relation to such neuropsychiatric disorders.

On this basis we created a transgenic rat model that via pronuclear microinjection overexpresses the DAT gene (Fig. 1). Neurobiological and behavioral studies were conducted on adult male hemizygous DAT-transgenic rats (*DAT-tg*) ubiquitously overexpressing DAT in the corticostriatal and associated networks.

## Results

### DAT and DRD1/2 receptor expression

**Figure 1:**
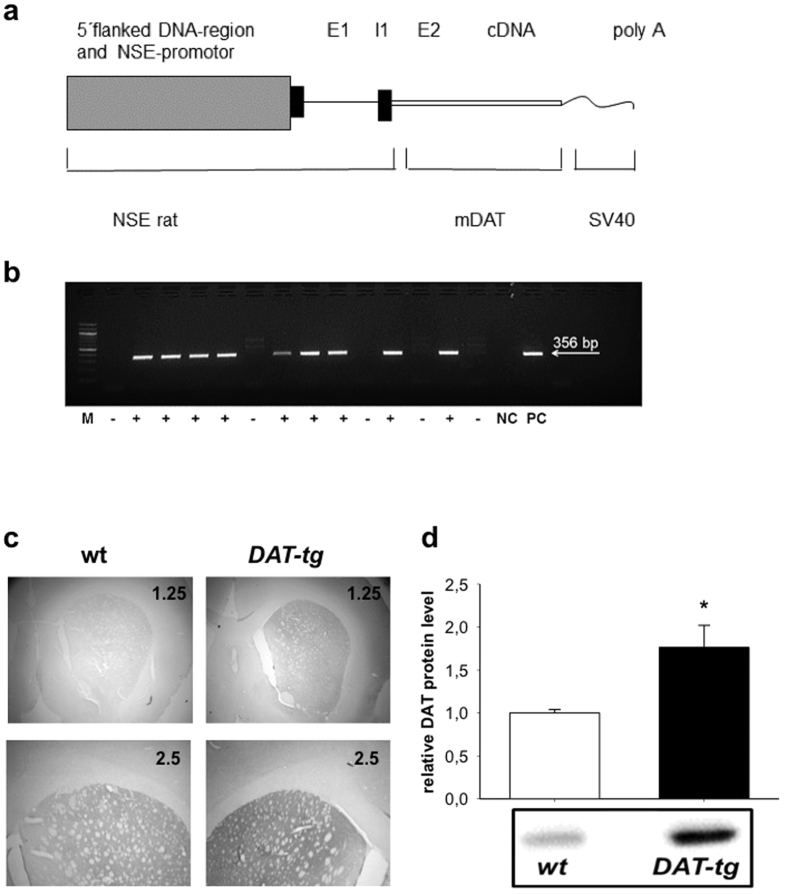
Generation of *DAT-tg* rats. **(a)** Schematic representation of the 4-kb DNA fragment used for the generation of the *DAT* transgenic rats. E1/2 = exon 1/2, and I1 = intron 1 of NSE, m*DAT* = murine *DAT* sequence, SV40 = Simian virus 40. **(b)** Representative *DAT* PCR products from *wt* (−) and *DAT-tg* (+) rats. M = marker, NC = negative control, PC = positive control, transgenic band = 356 bp. One founder line was used for the study. Here one litter from this generation is shown. **(c)** Representative coronal sections of immunohistochemical stain of DAT expression for wt (left) and DAT-tg (right). **(d)** *DAT* Western blot analysis of striatal tissue from *wt* (n = 5) and *DAT-tg* rats (n = 10).

**Figure 2:**
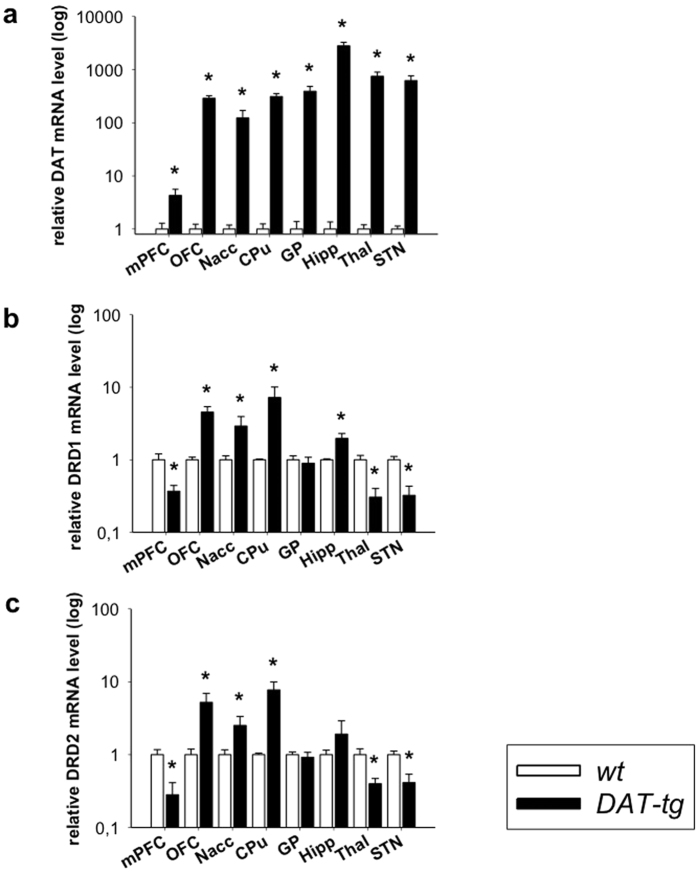
DAT and DRD1/2 receptor expression. **(a)** *DAT* qPCR analysis of corticostriatal and associated network regions in *wt* (n = 8) and *DAT-tg* rats (n = 7). **(b)** Dopamine receptor 1 (DRD1) and **(c)** Dopamine receptor 2 (DRD2) qPCR analysis of corticostriatal and associated network regions in *wt* (n = 8) and *DAT-tg* rats (n = 7). mPFC: medial prefrontal cortex, OFC: orbitofrontal cortex, Nacc: nucleus accumbens, CPu: caudate putamen, GP: globus pallidus, Hipp: hippocampus, Thal: dorsomedial thalamus, STN: subthalamic nucleus. All data are means ± s.e.m. Asterisk (*) indicates significant difference to *wt* rats with p < 0.05.

Western blot and qPCR were performed in order to assess the protein and mRNA expression levels of the dopamine transporter (DAT). qPCR was conducted to assess mRNA expression levels of the dopamine receptor 1 (DRD1), and dopamine receptor 2 (DRD2). Western blots showed that in comparison to *wt* rats, *DAT-tg* rats exhibited increased striatal protein-levels of the DAT transporter (striatum: T = −2.171, p = 0.05) (Fig. 1d). qPCR showed that in comparison to *wt* rats *DAT-tg* rats exhibited significantly increased DAT mRNA levels in the following areas: medial prefrontal cortex (mPFC (T = −2.588, p = 0.023)), orbitofrontal cortex (OFC (T = 9.161, p = 0.000)), nucleus accumbens (Nacc (T = −2.755, p = 0.016)), caudate putamen (CPu (T = −8.337, p = 0.000)), globus pallidus (GP (T = −4.579, p = 0.000)), hippocampus (Hipp (T-6.463, p = 0.001)), thalamus (Thal (T = −5.410, p = 0.000)), and subthalamic nucleus (STN (T = −4,589, p = 0.000)) (Fig. 2a). Further, *DAT-tg* rats exhibited increased DRD1 mRNA levels in the OFC (T = −3.534, p = 0.000), Nacc (T = −2.136, p = 0.029), CPu (T = −6.217, p = 0.036) and Hipp (T = −3.089, p = 0.009) and decreased DRD1 mRNA levels in the mPFC (T = 2.756, p = 0.016), Thal (T = 3.812, p = 0.002) and STN (T = 4.332, p = 0.000) (Fig. 2b). In a similar fashion, DRD2 receptors were upregulated in the OFC (T = −2.610, p = 0.022), NAcc (T = −1.917, p = 0.029) and CPu (T = −3.252, p = 0.006) whereas levels were downregulated in the mPFC (T = 3.246, p = 0.006), Thal (T = 2.646, p = 0.02) and STN (T = 3.414, p = 0.005) (Fig. 2c).

### Neurotransmitter contents and compensatory mechanisms

**Figure 3:**
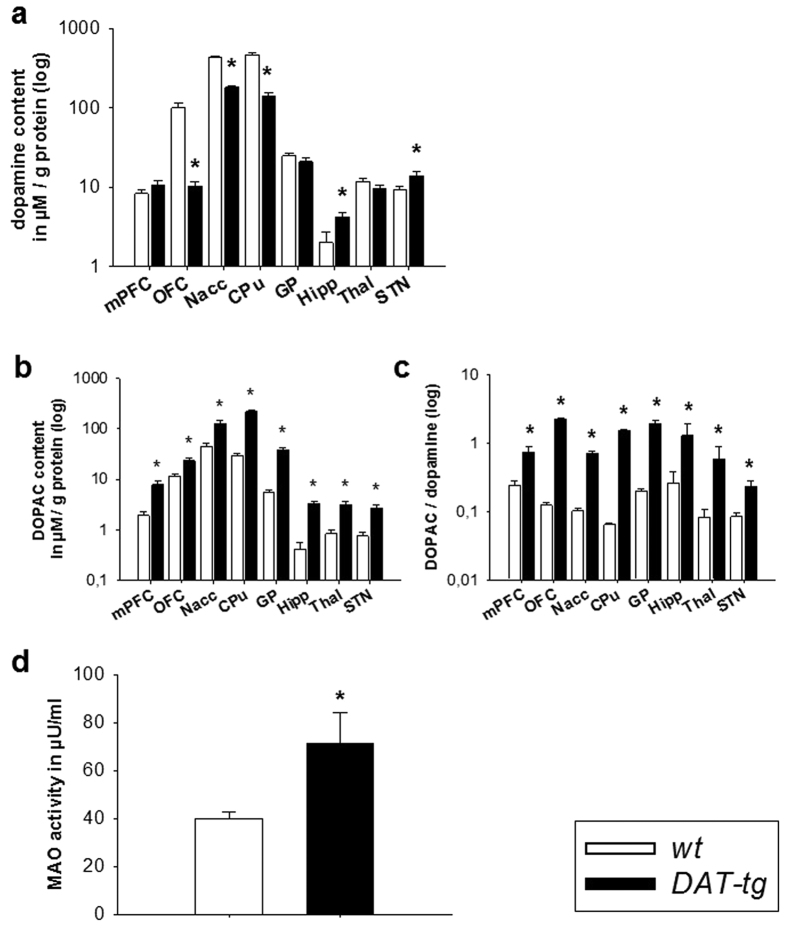
Neurotransmitter contents and compensatory mechanisms. Post mortem tissue **(a)** dopamine and **(b)** DOPAC contents as well as **(c)** dopamine turnover in corticostriatal and associated network regions in *wt* (n = 7) and *DAT-tg* rats (n = 10). mPFC: medial prefrontal cortex, OFC: orbitofrontal cortex, Nacc: nucleus accumbens, CPu: caudate putamen, GP: globus pallidus, Hipp: hippocampus, Thal: dorsomedial thalamus, STN: subthalamic nucleus. **(d)** Monoamine oxidase (MAO) activity of striatal tissues in *wt* (n = 8) and *DAT-tg* rats (n = 7). All data are means ± s.e.m. Asterisk (*) indicates significant difference to *wt* rats with p < 0.05.

**Figure 4:**
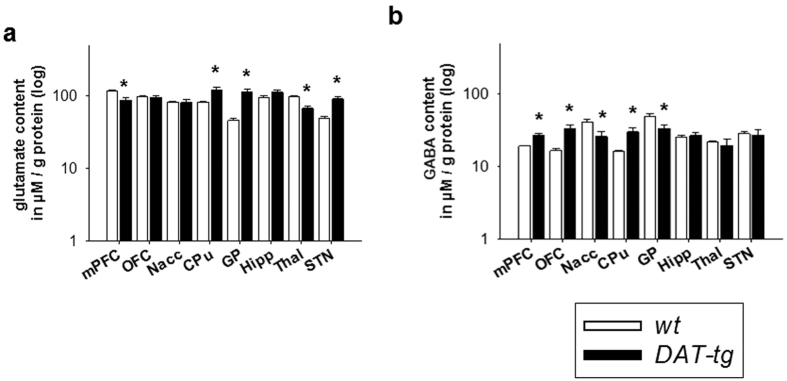
Glutamate and GABA contents. Neurochemical contents were examined in *wt* (n = 7) and *DAT-tg* rats (n = 10). Glutamate and GABA were measured in the medial prefrontal cortex (mPFC), orbitofrontal cortex (OFC), nucleus accumbens (Nacc), caudate putamen (CPu), globus pallidus (GP), hippocampus (Hipp), dorsomedial thalamus (Thal), and subthalamic nucleus (STN). All data are means ± s.e.m. Asterisk (*) indicates significant difference to *wt* rats with p < 0.05.

Post mortem HPLC was conducted to assess neurochemical contents of different neurotransmitter system. *DAT-tg* rats exhibited a decrease in tissue dopamine contents in the OFC (T = −7.504, p = 0.000), Nacc (T = −13,726, p = 0.000) and CPu (T = −14.611, p = 0.000), whereas an increase in dopamine was seen in the Hipp (T = 2.617, p = 0.020) and STN (T = 2.414, p = 0.029). With regards to metabolites and turnorver, *DAT-tg* rats exhibited increased DOPAC contents and dopamine turnover (DOPAC/dopamine) in the mPFC (DOPAC: T = 4.255, p = 0.000; turnover: T = 2.916, p = 0.011), OFC (T = 3.225, p = 0.006; turnover: T = 13.467, p = 0.000), Nacc (T = 4.391, p = 0.000; turnover: T = 7.542, p = 0.000), CPu (T = 9.134, p = 0.000; turnover: T = 19.314, p = 0.000), GP (T = 6.177, p = 0.000; turnover: T = 7.417, p = 0.000), Hipp (T = 5.884, p = 0.000; turnover: T = 1.35, p = 0.022), Thal (T = 4.009, 0.001; turnover: T = 1.505, p = 0.001) and STN (T = 4.503, p = 0.000; turnover: T = 2.962, p = 0.010) (Fig. 3a–c). For glutamate, *DAT-tg* rats exhibited increased contents in the CPu (T = 2.701, p = 0.016), GP (T = 4.934, p = 0.000) and STN (T = 4.113, p = 0.000) whereas a decrement was found in the thalamus (T = −4.574, p = 0.000). With respect to GABA, *DAT-tg* rats exhibited decreased contents in the Nacc (T = −2.665, p = 0.018) and GP (T = −2.231, p = 0.041) and increased contents in the mPFC (T = 2.962, p = 0.009), OFC (T = 3.161, p = 0.006) and CPu (T = 3.449, p = 0.004) (Fig. 4).

To investigate for possible compensatory mechanisms, monoamine oxidase (MAO) activity was assessed in striatal tissues. *DAT-tg* rats here exhibited a significant increase in total MAO activity as opposed to the *wt* rats (T = −2.470, p = 0.028) (Fig. 3d).

### Oscillatory activity

**Figure 5:**
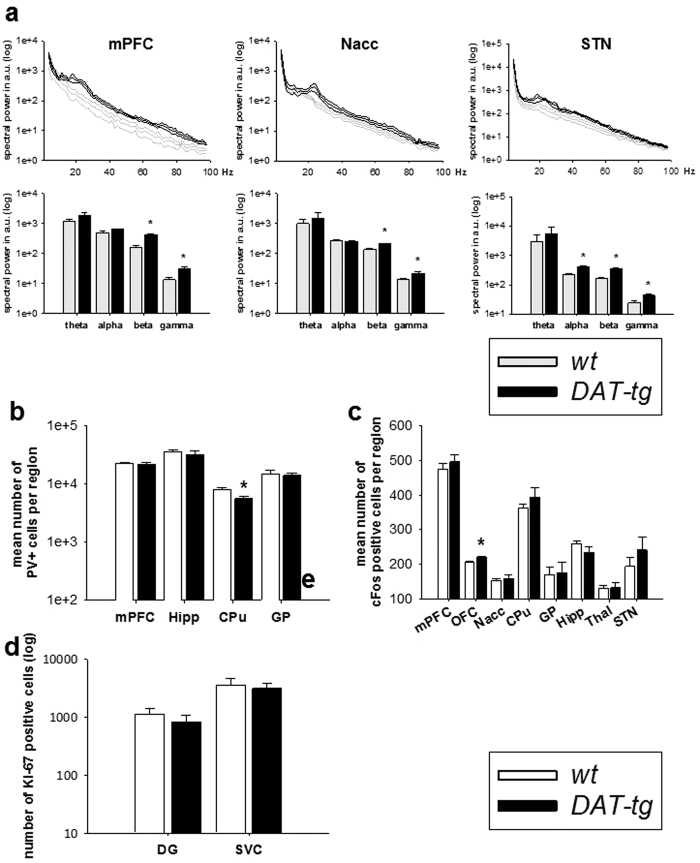
Oscillatory activity and immunostaining. **(a)** Oscillatory activity of the vmPFC, Nacc and STN in *wt* (n = 5) and *DAT-tg* rats (n = 7). Upper panel shows entire frequency range, lower panel shows mean values for the frequency bands: theta (4–8 Hz), alpha (8–12 Hz), beta (12–30 Hz), and gamma (30–100 Hz). **(b)** Immunohistochemical cell counts of parvalbumin expressing (PV+) cells of the medial prefrontal cortex (mPFC), hippocampus (Hipp), caudate putamen (CPu) and globus pallidus (GP) in *wt* (n = 12) and *DAT-tg* rats (n = 11). (**c)** c-Fos expressing cells on representative slices of the mPFC, orbitofrontal cortex (OFC), nucleus accumbens (Nacc), CPu, GP, Hipp, thalamus (Thal) and subthalamic nucleus (STN) in *wt* (n = 8) and *DAT-tg* rats (n = 8). **(d)** Immunohistochemical cell counts of Ki67 expressing cells in the neurogenic zones of the hippocampus (dentate gyrus, DG) and the subventricular zone (SVZ) in *wt* (n = 9) and *DAT-tg* rats (n = 9).

Oscillatory activity within the vmPFC, Nacc and STN was investigated via *in vivo* electrophysiological recording. The assessed frequency bands included: theta (4–8 Hz), alpha (8–12 Hz), beta (12–30 Hz), and gamma (30–100 Hz). Results show that in comparison to *wt* rats *DAT-tg* rats exhibited increased alpha, beta and gamma activity within the STN (alpha: T = −8.667, p = 0.000; beta: T = −8.972, p = 0.000; gamma: T = −2.781, p = 0.006) as well as increased beta and gamma activity within the mPFC (beta: T = −6.701, p = 0.000; gamma: T = −3.389, p = 0.000) and Nacc (beta: T = −3.723, p = 0.000; gamma: T = −2.594, p = 0.01) (Fig. 5a).

### Immunostaining

Immunostaining of parvalbumin expressing (PV)+ interneurons, c-Fos expressing nuclei and Ki67 expressing cells was conducted to investigate for possible cellular changes reflecting altered network activity. Results show that *DAT-tg* rats exhibited a significant reduction of PV+ cells specifically in the CPu as opposed to *wt* rats (T = 3.228, p = 0.004) (Fig. 5b). Further, *DAT-tg* rats exhibited a significant increase in cFos expressing cells specifically in the OFC as compared to *wt* rats (T = −2.884, p = 0.011) (Fig. 5c). No significant difference was found for Ki67 expression between *DAT-tg* and *wt* rats (Fig. 5d).

### Structural analysis of brain volumes

**Figure 6:**
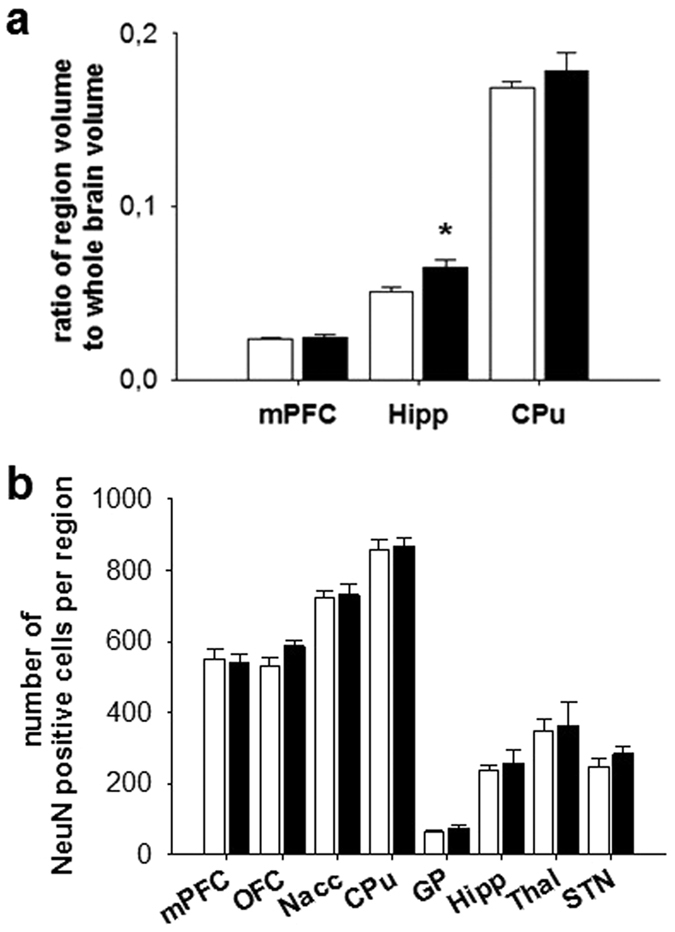
Structural analysis. **(a)** Volumes of the medial prefrontal cortex (mPFC), hippocampus (Hipp) and caudate putamen (CPu) relative to whole brain volumes as derived from MRI scans in *wt* (n = 6) and *DAT-tg* rats (n = 4). **(b)** NeuN expressing cells on representative slices of the mPFC, orbitofrontal cortex (OFC), nucleus accumbens (Nacc), CPu, globus pallidus (GP), Hipp, thalamus (Thal) and subthalamic nucleus (STN) in *wt* (n = 7) and *DAT-tg* rats (n = 5). All data are means ± s.e.m. Asterisk (*) indicates significant difference to *wt* rats (p < 0.05).

The whole brain volume and the volumes of the mPFC, Hipp, and CPu were assessed using structural MRI. *DAT-tg* rats exhibited a significant increase in Hipp volumes as compared to the *wt* rats (T = −3.326, p = 0.01) alongside unaltered whole brain volumes (Fig. 6a). NeuN immunostaining further revealed no difference between *DAT-tg* and *wt* rats (Fig. 6b).

### General behavioral assessment

**Figure 7:**
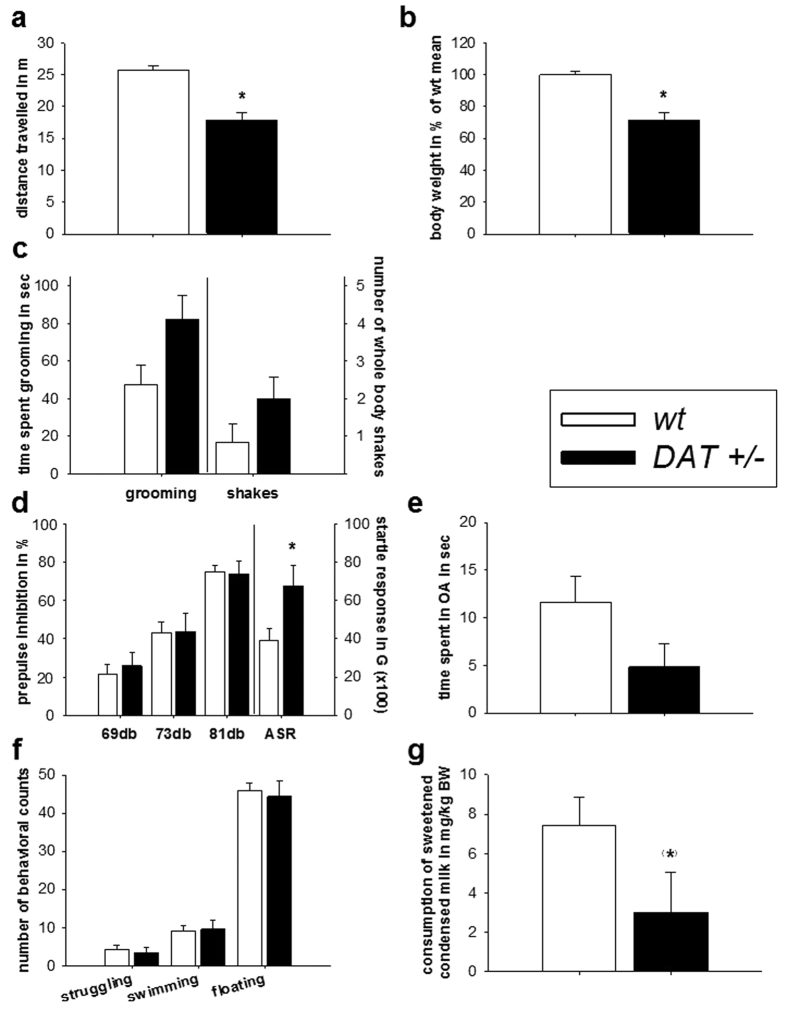
General behavioral assessment. General assessment of hemizygote *DAT* transgenic (*DAT-tg*) rats in comparison to *wt* rats. **(a)** *wt* and *DAT-tg* rats were weighed across lifespan. Body weight of *DAT-tg* rats was analyzed relative to body weight of age-matched *wt* rats. **(b)** Locomotion was analyzed as the total distance travelled on an open field over 30 min in each n = 10 *wt* and *DAT-tg* rats. **(c)** To study repetitive behavior upon stress-exposure, each n = 7 *wt* and *DAT-tg* rats were placed within the chambers used for prepulse inhibition (PPI) test and exposed to unpredictable acoustic stimuli. **(d)** Sensorimotor gating and arousal was analyzed in the PPI paradigm in *wt* (n = 14) and *DAT-tg* rats (n = 7). **(e)** Anxious behavior was measured in the elevated-plus-maze in *wt* (n = 15) and *DAT-tg* rats (n = 6). **(f)** In the forced swim test, the amount of time spent on struggling, swimming and floating was analyzed in *wt* (n = 14) and *DAT-tg* rats (n = 7). **(g)** In the sucrose consumption test the amount of sweetened condense milk consumed relative to body weight (BW) was measured in *wt* rats (n = 16) and *DAT-tg* rats (n = 7). All data are given as mean ± s.e.m. Asterisks (*) indicates significant difference between groups with p < 0.05.

*Wt* and *DAT-tg* rats were weighed across lifespan and body weights of *DAT-tg* rats were analyzed relative to body weight of age-matched *wt* rats. T-Test revealed *DAT-tg* rats to have significantly decreased body weights in comparison to *wt* rats across lifespan (T = 6.801, P = 0.000) (Fig. 7a). Figure 7b locomotion was analyzed as the total distance travelled on an open field over 30 min. T-Test revealed *DAT-tg* rats to travel significantly less than *wt* rats (T = 5.745, P = 0.001) (Fig. 7b). Figure 7a to study repetitive behavior upon stress-exposure, rats were exposed to unpredictable acoustic stimuli. T-test revealed *DAT-tg* rats to show a tendency towards more grooming (T = −2.070, P = 0.063) when compared to *wt* rats, but no significant increment in the number of whole body shakes (T = −1.527, P = 0.156) (Fig. 7c).

In the prepulse inhibition (PPI) paradigm, *DAT-tg* rats showed normal sensorimotor gating when compared to *wt* rats such that they expressed an unaltered suppression of the acoustic startle reflex (ASR) following acoustic stimuli of 69 db, 73 db, and 81db. However *DAT-tg* rats did show increased overall ASR reflecting hyper-arousal (T = −2.449, P = 0.024) (Fig. 7d). In the elevated-plus-maze and the forced swim test, no difference were found between *DAT-tg* rats and *wt* rats (Fig. 7e,f). In the sucrose consumption test *DAT-tg* rats showed when compared to *wt* rats a tendency to increased anhedonia as expressed in a reduced consumption of sweetened condense milk (T = 1.659, P = 0.071) (Fig. 7g).

### Repetitive behavior analysis

**Figure 8:**
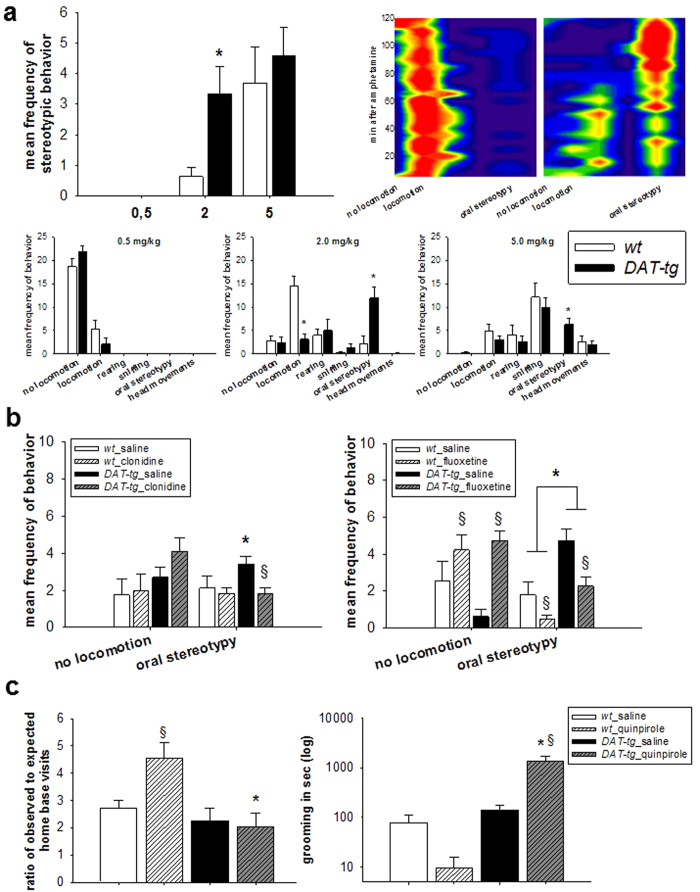
Repetitive behavior analysis. **(a)** Upper panel left: Repetitive behavior induced by d-amphetamine (0.5, 2.0 and 5.0 mg per kg body weight (BW)). Upper panel right: In *DAT-tg* rats, repetitive behavior evolves 80–120 min after d-amphetamine (2 mg /kg BW). The *wt* rats display hyperlocomotion throughout the period (120 min). Hot colors (red) indicate presence and cold colors (blue) absence of behavior. Lower panel: dose-dependent effects of d-amphetamine. **(b)** Clonidine effects (left) and fluoxetine (right) on locomotion and oral stereotypy following d-amphetamine (2 mg/kg BW) in *wt* (clonidine: n = 10; fluoxetine: n = 9) and *DAT-tg* rats (clonidine: n = 9; fluoxetine. n = 8). **(c)** Effects of quinpirole (QNP) on compulsive checking (left) and grooming behavior (right) in *wt* (n = 10) and *DAT-tg* rats (n = 10). All data are means ± s.e.m. Asterisk (*) indicates significant difference to *wt* rats, paragraph (§) indicates treatment effect (p < 0.05).

Repetitive behavior was assessed following the application of amphetamine (0.5 mg/kg, 2.0 mg/kg, and 5.0 mg/kg body weight (BW)) and saline over three consecutive days. To diminish the possibility of amphetamine-sensitization, dosages were applied in a randomized fashion. The assessed behavior included: no locomotion, locomotion, excessive rearing and sniffing as well as oral stereotypy and head movements. Administration of 0.5 mg/kg amphetamine was ineffective in both strains and administration of 5.0 mg/kg amphetamine induced repetitive behavior in both, *wt* and *DAT-tg* rats. Upon administration of 2.0 mg/kg amphetamine, *DAT-tg* rats exhibited a significant increase in repetitive oral movements (T = −3,545, p = 0.003), which effectively emerged 80–120 min after injection, whereas *wt* rats exhibited hyper-locomotion throughout the observation period (T = 4,718, P = 0.000) (Fig. 8a).

The effect of clonidine (0.01 mg/kg BW) and fluoxetine (20 mg/kg BW) versus saline on amphetamine (2 mg/kg BW) -induced behavior was assessed with respect to general movement and oral stereotypy. Same animals were exposed to drug administrations over a period of three consecutive days, with dosages applied in a randomized fashion. For the effect of clonidine on oral stereotypy, ANOVA revealed a significant effect for the factor phenotype (F = 6,598, p = 0.019) and a significant interaction (F = 6.887, p = 0.018). Subsequent post hoc analysis revealed that untreated *DAT-tg* rats exhibited significantly more repetitive behavior than untreated *wt* rats (p < 0.05) and that clonidine significantly reduced repetitive behavior in *DAT-tg* rats (p < 0.05). With regards to the effects of clonidine on locomotion, no significant effect was found. The effect of fluoxetine on oral stereotypy showed a significant effect for the factors phenotype (F = 27.061, p = 0.000) and treatment (F = 10.382, p = 0.006) with *DAT-tg* rats displaying significantly more repetitive behavior than *wt* rats and fluoxetine reducing it in both, *wt* and *DAT-tg* rats. With regards to the effect of fluoxetine on locomotion, a significant effect of treatment was found (F = 15.127, p = 0.001) (Fig. 8b) such that fluoxetine reduced locomotion in both, wt and *DAT-tg* rats.

The effect of quinpirole (0.5 mg/kg BW) and saline on compulsive checking and grooming was assessed using the following groups: *wt* + saline, *wt* + quinpirole, *DAT-tg* + saline, *DAT-tg* + quinpirole. For compulsive checking, a significant effect was found for phenotype (F = 11.464, p = 0.003) as well as a significant interaction across the factors phenotype and treatment. (F = 5.283, p = 0.032). Subsequent post hoc analysis revealed that quinpirole treated *wt* rats exhibited significantly more compulsive checking behavior as compared to untreated *wt* (p < 0.05) and quinpirole treated *DAT-tg* rats (p < 0.05). For grooming, a significant effect was found for both factors (phenotype: F = 22.960, p = 0.001; treatment: F = 17.091, p = 0.003) as well as a significant interaction (F = 21.278, p = 0.002) (Fig. 8b). Figure 8c following up on these effects, post hoc analysis revealed that in *DAT-tg* but not *wt* (p < 0.05) quinpirole significantly increased grooming when compared to saline conditions (p < 0.05).

## Discussion

Our results show that overexpression of the DAT induces multiple neurobiological and behavioral deficits that have also been observed in repetitive disorders.

Involuntary repetitive movements have shown to worsen under stress and upon amphetamine challenge. Such accentuated susceptibility to amphetamine has been reported for TS and differentiates this condition from obsessive-compulsive disorders (OCD), a further disorder belonging to the repetitive spectrum^9^. In terms of pharmacotherapy, the alpha-adrenergic and imidazoline receptor agonist α- clonidine serves as first line treatment due to its efficacy and tolerability^10^,^11^.

In rats, a typical expression of repetitive movements is grooming^12^. We here report, that *DAT-tg* rats showed increased grooming upon stress exposure. Upon d-amphetamine administration, both *wt* and *DAT-tg* rats developed repetitive behavior^13^. However, *DAT-tg* rats developed repetitive behavior already at amphetamine dosages ineffective in *wt* rats suggesting a susceptibility to amphetamine. This low-dose amphetamine induced repetitive behavior manifested over time with maximal expression 80–120 min after drug administration. It consisted of fragmented grooming patterns of face and paws that rarely continued into a full-body grooming syntax, and was associated with a typical motor confinement. Interestingly, this particular behavior was also the dominant behavior observed upon chronic intermittent application of the DRD2/DRD3 agonist quinpirole, which in *wt* rats induced compulsive checking behavior as previously reported^14^,^15^,^16^. Despite increased arousal and a tendency to anhedonia *DAT-tg* rats displayed intact sensorimotor gating, and scored normal in anxiety- and depression-associated paradigms. All together this suggests that behavioral abnormalities in *DAT-tg* rats are largely restricted to repetitive behavior symptomatology.

Testing pharmacotherapy, we found that clonidine specifically reduced repetitive behavior in *DAT-tg* rats whereas the serotonin reuptake inhibitor (SSRI) fluoxetine did not selectively affect repetitive behavior in *DAT-tg* rats. As expected, fluoxetine decreased locomotion in both phenotypes^17^. Clonidine is known to alleviate tics in TS whereas SSRI agents have been shown to ameliorate repetitive symptoms in OCD but not in TS.

The potential utility of *DAT-tg* rats in the context of repetitive disorder research is further supported by the neurobiological investigations of this study. TS has previously been associated with increased and decreased dopamine receptor availability^6^,^9^,^18^,^19^ and dopamine contents^5^,^18^,^20^,^21^,^22^. We found that ubiquitously induced DAT overexpression induced a region specific pattern of up- and downregulation. *DAT-tg* rats showed relative overexpression of DRD1 + DRD2 in the OFC, CPu and Nacc. This was further paralleled by increased striatal MAO enzymatic activity, previously linked to TS^23^. Increased MAO activity leads to increased dopamine turnover, resulting in decreased in dopamine levels. In contrast, DRD1 + DRD2 expressions were downregulated in the mPFC, Thal and STN and dopamine contents were reduced in the Thal and STN, which suggests a reciprocal regulation of dopamine receptor expression and tissue dopamine contents^24^.

In TS patients, an altered balance between GABAergic cells and glutamatergic projections is associated with abnormal corticostriatal circuit activity^2^. This imbalance is thought to result from reduced numbers of GABAergic parvalbumin expressing (PV+) interneurons. In accordance with that *DAT-tg* displayed region-specific increments and decrements in GABAergic and glutamatergic contents in the corticostriatal circuit. Further, we found a reduction of PV+ interneurons in *DAT-tg* rats as compared to *wt* rats. In line with clinical data^25^,^26^, this reduction was restricted to the CPu. Striatal PV+ interneurons coordinate striatal activity by increasing medium spiny neurons’ (MSN) firing threshold in response to cortical inputs^25^,^27^. Loss of PV+ interneurons found in TS patients is suggested to lead to MSN hyperactivity^25^,^26^. Both MSN and PV+ cells express dopamine receptors and depending on the membrane-potential are susceptible to dopaminergic activation^27^,^28^. The excessive depolarized state of MSNs facilitates the effect of dopamine on MSNs, which further reinforces their hyperactivity. As such, abnormalities in the striatal PV+ interneuron and dopamine systems may together induce an excessive activation of the cortico-striato-thalamic circuit leading to repetitive behavior^28^,^29^.

Further linkages of *DAT-tg* to repetitive disorders were gained by studies into neuronal cellular and population activity. *DAT-tg* rats exhibited upregulation of c-Fos in the OFC. Increased OFC activity is observed in patients with repetitive disorders^29^,^30^. *DAT-tg* rats further displayed increased beta and gamma oscillations in the mPFC, Nacc and STN and increased alpha oscillation in the STN. Beta activity in the STN is associated with movement abnormalities and inversely regulated by mesostriatal dopamine^31^. Alpha and gamma activity has been associated with spontaneous tic exertion and TS^32^. In general, alterations in LFP oscillatory activity are proposed as biomarkers of dopamine dysfunction^31^ and neuro-psychiatric disorders^33^.

Ectopic DAT overexpression has previously been linked to neurotoxic events including oxidative stress and neuronal loss^34^,^35^,^36^. To explore whether DAT overexpression induced neuropathological changes in the *DAT-tg* rat, we measured the volume of the mPFC, the striatum and the hippocampus as these areas in the DAT-tg rat displayed both ectopic DAT expression and dopaminergic input but showed differential effects of DAT-overexpression on DA contents. MRI data displayed no atrophy in either the mPFC or the CPu but increased Hipp volumes in DAT-tg as compared to control rats. Increased Hipp volumes have been suggested to constitute a compensatory response in TS^37^. Further immunostaining of the neuron-specific marker (NeuN) revealed no difference between the phenotypes, which stresses the notion that ubiquitous overexpression of DAT does not induce neurotoxicity in the *DAT-tg* rat.

Our findings support the hypothesis that the DAT may constitute one important key component in repetitive pathophysiology and that DAT overexpression might be of relevance for further comprehension of neurobiological mechanisms underlying neuropsychiatric disorders.

## Experimental Procedures

### Rats

Rats were housed in a temperature- and humidity-controlled vivarium with a 12-h light dark cycle (lights on 06:00 a.m.) with food and water available ad libitum. The study was carried out in accordance with the European Communities Council Directive of 22th September 2010 (2010/63/EU) and after approval by the local ethic committees (Senate of Berlin and Regierungspräsidium Dresden). All efforts were made to reduce animal suffering and number of animals used.

#### Preparation of the construct

The pcDNA3-murine dopamine transporter^38^ (mDAT) cDNA-vector was kindly provided by Heinz Bönisch (Institute of Pharmacology and Toxicology, University of Bonn, Germany) (Fig. 1a). It contains the full coding region of the mDAT cDNA and has been cloned by PCR with a sense primer derived from the partial mDAT gene sequence and an antisense primer deduced from the rat DAT cDNA. In this construct the CMV promoter was replaced by the rat NSE promoter isolated from the pNSE-Ex4 vector comprising 2.6 kb of 5′-untranslated sequence plus exon 1, intron 1, and 6 bp of exon 2 but not the ATG start codon of NSE. Sequencing was performed by the University of Calgary DNA Sequencing Laboratory to confirm the sequence of the construct. The construct consisting of NSE promoter, mDAT coding sequence, and bovine growth hormone polyadenylation sequence was excised from the pcDNA3 vector with NruI/NaeI, purified by agarose gel electrophoresis and gel extraction using the QIAquick gel extraction kit and used for microinjection. The NSE promoter was chosen for the expression of DAT to avoid probable unpredictable effects due to increment in monoamine in dopaminergic nerve endings.

***Generation of transgenic rats*** was conducted as reported previously^39^. Briefly, immature female Sprague-Dawley (SD) Hanover rats (28 to 35-day-old from Janvier Labs, France) were induced to superovulate by intraperitoneal (i.p.) injection of PMSG (15 IU, Intervet) and hCG (30 IU Sigma). Thereafter, rats were mated with fertile males and 24 h later sacrificed to collect fertilized eggs. The DNA construct was microinjected into the pronucleus of zygotes^40^,^41^. Eggs were cultured for two hours and the surviving DNA-injected zygotes were transferred into the oviducts of pseudopregnant SD recipients at the day the vaginal plug was detected. Integration of the transgene was determined by transgene-specific PCRs with genomic DNA isolated from tail biopsies of the offspring after weaning (Fig. 1b). Neurobiological and behavioral studies were conducted on adult male hemizygous *DAT*-transgenic rats (*DAT-tg*) ubiquitously overexpressing *DAT* in the corticostriatal and the associated networks. Wildtype (*wt*) rats served as controls. Immunohistochemical staining of DAT expression was carried out for wt and DAT-tg rats (Fig. 1c).

### Tissue processing

For Western blotting (WB), quantitative real time PCR (qPCR), and post mortem HPLC, and MAO activity assay, rats were decapitated and micropunches were taken bilaterally from 0.5–1 mm thick brain slices from the medial prefrontal cortex (mPFC), orbitofrontal cortex (OFC), thalamus (Thal), hippocampus (Hipp), nucleus accumbens (Nacc), caudate putamen (CPu), globus pallidus (GP) and subthalamic nucleus (STN) as described previously^42^. The total RNA and protein was extracted using the NucleoSpin RNA/Protein-Kit (Machery-Nagel, Düven, Germany). For immunostaining, rats were transcardially perfused, brains postfixed in 4% paraformaldehyde and cryosectioned in 40-μm serial coronal frozen sections.

#### Western blotting

Protein concentrations were determined using a Nanodrop Spectrophotometer (peqlab) (UV 280 nm). Samples (pooled Nacc and CPu specimen only) were loaded alongside Precision Plus Protein Kaleidoskope Standards (Bio-Rad), subjected to discontinuous electrophoresis on 10% SDS-polyacrylamide gels and then transferred onto PVDF membranes (Roth) by electroblotting. Membranes were first incubated in SuperBlock T20 (TBS) Blocking Buffer (Lifetechnologies) at room temperature for 1 hour, and then incubated at 4 °C overnight with the primary antibodies: anti-DAT (1:200 dilution, Santa Cruz, sc-14002). A ß-actin antibody (1:800 dilution, Cell Signaling. 4967S) was used for internal control. Membranes were washed and incubated with horseradish peroxidase-conjugated secondary antibodies (1:5000 dilution, Amersham, ECL Rabbit IgG, HRP-linked whole antidbody: GE Healthcare Life Science NA934) at room temperature for one hour. For repeated analysis, membranes were stripped with Restore™ Plus Western Blot Stripping Buffer (Thermoscientifc). Detection of immunoreactive bands was conducted using the Western lighting plus enhanced chemiluminescence (ECL) reagent (PerkinElmer) on a cooled charge-coupled device camera (FLI Proline PL09000, PA, USA). Images were processed using the Image J software.

#### qPCR

RNA concentrations were determined using a Nanodrop Spectrophotometer (peqlab). cDNA was synthesized using the High Capacity RNA-to-cDNA Kit (Lifetechnologies). TaqMan qPCR was performed with StepOne Real-Time PCR System (Lifetechnologies) using TaqMan fast advanced master mix (Lifetechnologies). The following TaqMan Gene Expression assays (Lifetechnologies) were used: DAT (Rn00562224_m1), DRD1 (Rn 03062203_s1), and DRD2 (Rn01418275_m1). CT values were normalized to the house keeping gene GFAP (Rn00566603_m1, Lifetechnologies), fold change was calculated using the ∆∆CT method.

#### Monoamine oxidase activity assay

For assessing monoamine oxidase (MAO) activity, CPu punches were homogenized by ultrasonication in 70 ul assay buffer of a fluorometric assay kit (biovision K795–100). MAO activity was assessed according to the user manual.

#### Post mortem HPLC

Post mortem HPLC was conducted as described previously^42^. Dopamine and its metabolite DOPAC were separated on a column (ProntoSil 120-3-C18-SH; Bischoff Analysentechnik und -geräte GmbH, Germany) and electrochemically detected (Chromsystems Instruments & Chemicals GmbH, Germany). Glutamate and GABA were precolumn-derivatized with *o*-phthalaldehyde-2-mercaptoethanol, separated on a column (ProntoSil C18 ace-EPS) and detected by their fluorescence at 450 nm after excitation at 330 nm.

#### Immunostaining

Free-floating sections were stained with antibodies against Ki67 (1:500, Novocastra, NCL-Ki67p), NeuN (1:5000, Millipore MAB377), DAT (1:50, Millipore AP1569P), Parvalbumin (PV+, 1:500, Antikörper-online, ABIN1742405), c-Fos (1:100, Santa Cruz, sc-52) and detected with goat-anti-rabbit biotinylated secondary antibodies (1:1000, Vector Laboratories, BA1000). For PV+ immunostaining, one-in-twelve series from the rostral-caudal extent of the mPFC, Hipp, CPu and GP and for Ki67 immunostaining one-in-twelve series from the Hipp and the subventricular zone (SVC) were analyzed. For c-Fos and NeuN immunostaining, the number of positive nuclei that fell within a 0.5 × 0.5 mm area (x 2,5 objective) in the mPFC, OFC, Thal, Hipp, Nacc, CPu, GP and STN was counted from one-in-twelve series sections from the rostral-caudal extent of the respective regions^43^. A representative picture of the DAT transporter was obtained.

### MRI

MRI was performed using a 7 Tesla rodent scanner (Pharmascan 70/16, Bruker BioSpin,Ettlingen, Germany) and a ^1^H-RF quadratur-volume resonator with an inner diameter of 20 mm on *ex vivo* brains. Data acquisition and image processing were carried out with the Bruker software Paravision 5. All brains had been perfused and snap frozen in methylbutan. 24 hours prior to the scan, all brains were placed in phosphate-buffered saline (PBS) and stored at 4 °C, to allow for the defrosting of the brains. On the day of MRI acquisition, rat brains were placed in a 15 ml Falcon tube containing PBS–with the anterior-posterior axis of the brain co lining with the long axis of the tube. For imaging the whole brain a T2-weighted 2D turbo spin-echo sequence was used (imaging parameters TR/TE = 5980.3/36 ms, rare factor 8, 4 averages, 50 axial slices with a slice thickness of 0.5 mm, field of view of (FOV) 20.59 × 20.59 mm, matrix size 256 × 256). Brain structure volume was estimated as the mean magnitude of regions of interest (ROI) using ImageJ software.

### Electrophysiology

Local field potentials (LFPs) were recorded under urethane anesthesia (1.2 g/kg i.p., Sigma Aldrich, Germany) as descried previously^44^. Monopolar recording electrodes (polyimide insulated stainless steel, 0.125 mm, Plastics One, USA) were implanted ipsilaterally into the left mPFC, Nacc shell, and STN at the following coordinates with respect to bregma: mPFC: AP = 3.5, ML = 0.6, DV = −3.4, Nacc: AP = 1.2, ML = 1.8, DV = −8.1, STN: AP = −3.6, ML = 2.5, DV = −7.6^45^. Recordings were referenced against 1.2 mm steel screws affixed to the skull in close proximity to each recording electrode. Signals were bandpass filtered (0.05 Hz–300 Hz), amplified, sampled at 1 kHz and digitized using a programmable neuronal data acquisition system (Omniplex, Plexon, Texas, USA). Recordings were conducted over a period of five hours. Offline data from the mPFC were inspected visually to identify and analyze epochs (40–50 s) of robust activated synchronization states (AS) reflecting signals of awake behaving rats^46^. The same time segments identified to show robust AS in the mPFC were also used for analysis of LFPs from the Nacc shell. Power spectral densities of the LFP data segments were calculated by employing the Fast Fourier Transform function (Spike 2 Version 6 data analysis software; Hanning Window (1024 ms), 0.9766 Hz resolution). Frequency spectrum was divided into four EEG bands: theta (4–8 Hz), alpha (8–12 Hz), beta (12–30 Hz), gamma (30–100 Hz). Power spectra were normalized to total power between 103–147 Hz and 153–197 Hz. Power was averaged across the specific frequency bands and further expressed in arbitrary units (a. u.). Correct electrode tip placements were histologically verified.

### Behavioral analysis and drug treatment

#### Amphetamine-Induced stereotypy

Testing took place in individual testing boxes (50 × 50 × 50) composed of 4 identical Plexiglas walls. Boxes were visually isolated from each other by an opaque screen. Experiments were performed over three consecutive days, during which animals were subjected to the three different dosages of d-amphetamine (i.p 0.5 mg/kg, 2.0 mg/kg or 5.0 mg/kg, dissolved in 0.9% saline at a volume of 1.0 ml/kg, Sigma Aldrich, Germany) in a cross over design. On testing days and prior to injection animals were habituated to the testing boxes for 20 min. Following injection, animals were immediately placed back into the testing boxes and behavior was recorded for 120 min. For analysis, the 120 min test was divided into 5-min segments and the most prominent behavior was scored for each segment. Behavioral scoring was based on an adapted version of the scoring protocol employed by Carter *et al*.^47^ dividing behavioral expression into (i) limited exploratory activity with discontinuous sniffing/grooming/rearing (no locomotion), (ii) constant exploratory activity with discontinuous sniffing/grooming/rearing (locomotion), (iii) continuous rearing (rearing), (iv) continuous sniffing (sniffing), (v) continuous biting, gnawing or licking (oral stereotypy), (vi) continuous head swaying/head bobbing (head movements).

#### Amphetamine and clonidine/fluoxetine treatment

Testing took place in testing boxes as described above. Experiments were performed over two testing days 72 h apart, during which animals were randomly assigned to treatment (clonidine/fluoxetine) or control (saline) conditions in a cross over design. On both testing days, all animals were initially habituated to testing boxes for 20 min after which they were injected with amphetamine (2.0 mg/kg, dissolved in 0.9% saline at a volume of 1.0 ml/kg, Sigma Aldrich, Germany), placed back into the testing boxes and video recorded. 50 min after amphetamine injection, animals were injected with clonidine (i.p 0.01 mg/kg, dissolved in 0.9% saline at a volume of 10 ml/kg, Sigma-Aldrich, Germany), fluoxetine (20 mg/kg, dissolved in 0.9% saline at a volume of 1.0 ml/kg, Hexal, Germany) or saline after which they were placed back into the testing boxes. Behavior was analyzed for the period of most prominent expression of oral stereotypy in drug-free conditions, i.e. 80–120 min post amphetamine application. For analysis, the 40 min test period was divided into 5-min segments and the most prominent behavior was scored for each segment as described above.

#### Quinpirole induced repetitive behavior

Rats treated chronically with the dopamine D2/D3 receptor agonist quinpirole (QNP) develop compulsive-like behaviors that resemble compulsive checking behavior of OCD patients^15^. Rats were injected subcutaneously twice weekly for a total of 10 injections with either saline or QNP hydrochloride (0.5 mg/kg body weight, 0.5 mg/ml 0.9% NaCl, Sigma® Aldrich, Germany). Fifteen minutes after each injection, animals were placed in an open field that consisted of a glass table (140 × 140 and 20 cm high) subdivided into 25 rectangles (locales) and equipped with 4 plexiglas boxes at fixed locations. The 10^th^ session, when QNP treated rats are known to display compulsive checking behavior was videotaped and analyzed using tracking software (VideoMot 2 system, TSE, Bad Homburg, Germany). The following measures were assessed: (i) total distance traveled, (ii) total time of activity/inactivity, (iii) frequency of stops at each open field locale, (iv) mean duration of return time to a given locale, (v) mean stop duration at a given locale, (vi) total duration of stops at a given locale. The locale with the highest total duration of stops was individually defined as the home base and compulsive checking behavior was analyzed with reference to the HB. Compulsive checking is present if the rat meets the following three criteria: it returns to HB excessively often, excessively rapidly, and visits less places before returning to the HB. As repeated administration of QNP increases locomotion and since checking behavior requires locomotion, arithmetic was applied allowing the assessment of checking behavior relatively independent from locomotion. Specifically, for each rat individually, the expected rate of return to a locale was calculated by dividing the total number of visits in a session by the number of locales visited. Next, the ratio of observed to expected HB visits was calculated by dividing the number of visits to the HB with the expected rate of return to a locale. Additionally, the total time spent on grooming/oral stereotypy on the 10^th^ test day was calculated.

#### Startle stress response

Animals were exposed to unpredictable acoustic stimulus to investigate the effect of stressor-exposure on repetitive behaviour. Animals were placed in the chambers used for prepulse inhibition (PPI) test. The plastic enclosure used to restrain the rats during PPI testing was removed. The door was left open and a piece of clear Plexiglas was placed in front of the opening of the chamber to prevent the rats from escaping. Rats were acclimated to the box for 10 min, then a PPI protocol was initiated and run for 10 min, thereafter rats were left undisturbed for further 10 min^12^. The process was recorded and scored on playbacks. The total time spent on grooming as well as the number of whole body shakes during the 20 min after PPI protocol initiation was analyzed.

#### Prepulse inhibition of an acoustic startle response

Acoustic startle response (ASR) and PPI of the ASR was assessed using a standard startle chamber (SR-lab, San Diego Instruments). An adapted version of the general SR-LAB startle response user manual was applied. Animals were exposed to a 5 min acclimatization phase of white noise at 65 dB, followed by 5 initial startle stimuli (120 dB, each presented for 40 ms). The test session was pseudorandomized and composed of 40 startle stimuli presented either alone (120 dB for 40 msc), or proceeded by a pre-pulse of either 69, 73 or 81 dB for 20 ms, 100 ms before the startle. Each pulse or pre-pulse trial was separated by inter-trial intervals of a randomized duration ranging from 15–30 seconds, during which white background noise was presented (65 dB), leading to a total testing time of approximately 40 min. The animals’ startle reaction to the stimuli alone and to the pre-pulse trials was measured for 100 ms following the stimulus and amplitude as well as percentage decrease in startle response with pre-pulses (pre-pulse inhibition) was estimated^41^.

#### Elevated plus maze

Animals were placed in the center of an elevated plus maze (EPM, 42 × 42 cm, arm width: 23 cm), composed of two closed and two open arms. The animals were allowed to freely explore the maze for 5 min, while behavior was recorded via a web camera. The total time spent on open arms (OA, with both front- and hind paws placed on the arm) was determined^48^.

#### Forced swim test

Animals were conditioned to water-filled glass cylinders (depth of 30 cm, 25 °C) for 15 min 24 h prior to testing. The cylinders were visually isolated from each other by an opaque screen. On testing day, animals were placed in the cylinders for 5 min and behavior was recorded via a web camera. For behavioral analyses, the 5 min test was divided into 5-second segments and the most predominant behavior was determined per segment (struggling, swimming and floating behaviour)^48^.

#### Sucrose consumption test

48 h prior to testing, animals were habituated to the individual testing cages and bottles (containing water). 24 h thereafter, animals were habituated to the sweetend condensed milk (Nestlé, Milchmädchen gezuckerte Kondensmilch, (1:3)) for 30 min in their home cage and subsequently food restricted until time of testing (15 g per animal). On the day of testing, animals were placed in the individual cages with free access to the sweetend condensed milk for 15 min. Bottles were weighed before and after testing. The amount of sweetend condensed milk consumed normalized to individual body weight was calculated^48^.

### Blinding

Throughout the experiments best possible blinding was conducted. For video tracking during behavioral testing, animals were number-coded such that the experimenter was blinded to phenotype and treatment condition during later video analysis. The same system was applied to neurobiological analysis.

### Statistical analysis

Data are shown as means ± s.e.m. We used Student’s t test to calculate significant differences between wt and DAT-tg rats. We used two-way ANOVA with the factors phenotype (*wt, DAT+/*) and treatment (saline, QNP) for behavioral analysis of QNP induced repetitive behavior, and two-way ANOVA with repeated measure with the factors phenotype (*wt, DAT-tg*) and treatment (saline, clonidine/fluoxetine) for drug experiments followed by Holm-Sidak post hoc test if applicable. Significance was set at P < 0.05.

## Additional Information

**How to cite this article**: Hadar, R. *et al*. Rats overexpressing the dopamine transporter display behavioral and neurobiological abnormalities with relevance to repetitive disorders. *Sci. Rep.* **6**, 39145; doi: 10.1038/srep39145 (2016).

**Publisher's note:** Springer Nature remains neutral with regard to jurisdictional claims in published maps and institutional affiliations.
